# Early Exposure to Lesbian, Gay, Bisexual, Transgender, Queer (LGBTQ+) Medicine: Assessing Confidence and Comfort in Preclinical Medical Students

**DOI:** 10.7759/cureus.41834

**Published:** 2023-07-13

**Authors:** Kelsee K Zajac, Jenna Gunn, Rand El-Sharaiha, Dani Zoorob

**Affiliations:** 1 Department of Medical Education, University of Toledo College of Medicine and Life Sciences, Toledo, USA; 2 Department of Obstetrics and Gynecology, University of Toledo College of Medicine and Life Sciences, Toledo, USA; 3 Department of Obstetrics and Gynecology, Louisiana State University Health Sciences Center at Shreveport, Shreveport, USA

**Keywords:** diversity and equity in medicine, diversity, medical school education, lgbtq medicine, lgbtq

## Abstract

Background: Lesbian, Gay, Bisexual, Transgender, Queer (LGBTQ+) individuals face unique health challenges when compared to the general population. Physicians can play an integral role in either addressing these health inequities or further perpetuate discrimination. Despite the growing LGBTQ+ population in the United States and exposure during clinical care, many medical schools still lack an effective, standardized LGBTQ+ healthcare curriculum. Research has shown that when medical students receive exposure to LGBTQ+ healthcare topics, it results in superior quality of care. Considering the unique challenges LGBTQ+ individuals face, coupled with the perception medical students have of the current LGBTQ+ curriculum, and the positive impact LGBTQ+ education may have on patient care, there is a need for an effective and standardized LGBTQ+ curriculum in medical school education.

Objectives: The aim of this study was to assess the effectiveness of a two-hour interactive LGBTQ+ workshop at increasing confidence and comfortability in LGBTQ+ topics and healthcare education for preclinical medical students.

Methods: Twenty-five first- and second-year medical students participated in an optional two-hour interactive LGBTQ+ workshop. The first hour consisted of a lecture overviewing LGBTQ+ clinical medicine from a physician specializing in LGBTQ+ topics and care. The second hour was made up of four 15-minute stations. Students were split up evenly and rotated through these four stations consisting of: (1) a one-on-one standardized patient simulation, (2) discussion-based case scenarios, (3) an interactive seminar on transgender healthcare, and (4) a debriefing station. All facilitators and standardized patients were members of the LGBTQ+ community. Consenting participants were provided with a pre- and post-survey consisting of basic demographic questions, and 16 LGBTQ+ healthcare specific statements that they answered using a 7-point Likert scale.

Results: Fifteen of the 25 (60%) preclinical medical students completed all components of both the pre- and post-survey. 53.3% of the respondents were heterosexual, while 40% identified as being a part of the LGBTQ+ community. Survey results demonstrated a significant increase compared to the pre-workshop baseline in preclinical student comfort and confidence in 12 out of the 16 LGBTQ+ healthcare specific statements after completion of the workshop.

Conclusions: Our study suggests that focused education, such as through workshops, on LGBTQ+ topics can significantly increase preclinical student comfort and confidence when encountering LGBTQ+ clinical scenarios. In the future, we hope this workshop is implemented within our core medical school curriculum as a mandatory course to reach a wider audience. This workshop offers an efficient and effective model for other medical schools to implement to educate their medical students on LGBTQ+ healthcare topics.

## Introduction

From 2017 to 2020, individuals that identified as Lesbian, Gay, Bisexual, Transgender (LGBT) rose from 4.5% to 5.6% in the United States [1]. When compared to the general population, common health challenges faced by the Lesbian, Gay, Bisexual, Transgender, Queer (LGBTQ+) community include higher risk for mental health disorders, suicide, substance abuse, sexually transmitted infections, cancers, cardiovascular disease, and obesity [2]. These health challenges can be further perpetuated by a lack of trust among LGBTQ+ individuals in the U.S. healthcare system [3]. One survey of LGBTQ+ individuals from Colorado found that 65% of LGBT respondents and 85% of transgender respondents felt that there were not enough adequately trained health professionals who understand the nuances of LGBTQ+ health [4]. This thought, along with other social anxieties and fears surrounding gender identity and sexual orientation, may deter these patients from being open and honest about their sexual practices, gender identity, or sexual orientation, or deter them from even seeking medical care [5-7].

Several factors contribute to the healthcare challenges observed in the LGBTQ+ community. Physicians can play a role in addressing these health inequities. However, without proper training, healthcare professionals can also potentially further perpetuate discrimination inadvertently [8]. An aspect of these inequities originates from the lack of exposure to LGBTQ+ medicine in the early training of healthcare professionals. Despite the growing number of LGBTQ+ individuals in the United States, many medical schools still lack effective LGBTQ+ education included in their curriculum. In 2015, a survey sent to U.S. and Canadian medical students inquiring on the efficacy of their LGBTQ+ curriculum demonstrated 67% of students rating their education as, “fair or worse” [9]. Furthermore, a 2018 survey of New England medical students reported that of the 658 respondents, 80% felt either ‘somewhat competent’ or ‘not competent’ in providing medical treatment to LGBTQ+ patients [10].

One study found that when medical students had experiences and exposure to LGBTQ+ related healthcare, it resulted in superior quality of care for LGBTQ+ patients when compared to those with less experience [11]. Furthermore, a health survey of the LGBTQ+ community in Colorado found that patients who perceived their medical provider to be LGBT-friendly were more likely to participate in health-positive activities, such as seeing a primary care physician in the past six months or receiving the flu shot [4]. Although this data exists, not all medical school curricula provide students with the opportunity to practice clinical scenarios with standardized patients, nor offer a curriculum that leaves students feeling fully competent in LGBTQ+ healthcare related topics [12]. Medical students spend their first and second years of school learning the basic sciences. Years three and four are defined as “clinical years” and are spent applying this knowledge into clinical practice through patient interactions.

Considering the growing LGBTQ+ population, the current perception that medical students have of the current LGBTQ+ curriculum, and the positive impact proper training may have on clinical outcomes, the implementation of focused education for preclinical students should be prioritized. In this study, we aimed to assess whether a two-hour interactive workshop could increase preclinical medical student confidence and comfortability with LGBTQ+ topics and healthcare education.

## Materials and methods

The study was approved by the University of Toledo College of Medicine and Life Sciences (UTCOMLS) Institutional Review Board (IRB# 301176-UT). Twenty-five preclinical medical students at UTCOMLS participated in this study. A pre- and post-survey consisting of basic demographic questions and 16 LGBTQ+ healthcare specific statements were answered by consenting participants using a 7-point Likert scale on Qualtrics.

Structure and Content of Workshop

The University of Toledo College of Medicine and Life Sciences LGBTQ+ organization implemented a workshop focused on LGBTQ+ clinical health scenarios with preclinical medical students. The first hour was in lecture format from a guest physician specialized in LGBTQ+ clinical medicine. Topics discussed included an overview of terminology, sexual and gender identity, gender-inclusive language, health disparities prevalent within the community, barriers to care, population statistics, and different ways to create a safe and welcoming space for patients.

For the second hour, students were split up evenly into four groups and rotated through 15-minute stations. These stations consisted of: (1) a one-on-one standardized patient simulation, (2) discussion-based case scenarios, (3) an interactive seminar on transgender healthcare, and (4) a debriefing station. All facilitators and standardized patients participating were members of the LGBTQ+ community. The standardized-patient experience was dedicated to students interviewing an LGBTQ+ patient coming into the office for an annual check-up, obtaining a sexual history, and providing education on safe sex practices. The interview was followed by formative feedback from the standardized patient to the student. Case scenarios were led by facilitators ranging from common healthcare scenarios within the LGBTQ+ community to day-to-day dilemmas that sexual and gender minority people commonly face. The interactive seminar on transgender healthcare consisted of a mini-lecture with a brief overview of the different types of gender-affirming care, as well as specific healthcare disparities prevalent in the transgender community. Finally, the debriefing station allowed for open-ended discussion, along with facilitator-led questions to guide the conversation.

Statistical Analysis

A two-sample paired t-test was used to assess the statistical significance of the data obtained from the responses collected from the pre- and post-session surveys. Each statement was given a score between 1 and 7 (7 being extremely comfortable and 1 being extremely uncomfortable) based on the student’s level of comfort before and after the workshop. The mean score of the responses pre- and post-survey were compared to assess if there was an increase in comfort and confidence. A p-value of less than 0.05 was considered significant.

## Results

Participant Demographics

The workshop included 25 medical student participants. However, 15 of the 25 (60%) first- and second-year medical students consented to participate and completed all components of the pre- and post-workshop survey, where 66.7% (N=10) of the respondents were cis-gender females, versus 33.3% (N = 5) who were cis-gender males. The sample size for statistical analysis was 15. All respondents were preclinical medical students with the majority being first year medical students (73.3%, N=11) compared to second year medical students (26.7%, N=4). The majority of participants’ ethnicity was White/Caucasian (68.7%, N=10), followed by Asian (12.5%, N=2), Black/African American (12.5%, N=2), and Native American/Alaskan Native/Indigenous (6.3%, N=1). None of the respondents identified as being Hispanic. For sexual identity, 53.3% (N=8) of respondents were heterosexual, with 40.1% (N=6) identifying as being LGBTQ. One individual identified their sexuality as 'other'. The demographics of the participating students are further summarized in Table 1.

**Table 1:** Workshop Participant Demographics

Gender identity	Percentage (%)
Cis-gender Female	66.7
Cis-gender Male	33.3
Other	0
Year in Medical School	
1^st^ Year	73.3
2^nd ^Year	26.7
Ethnicity/Race	
Asian	12.5
Black/African American – Not Hispanic	12.5
Native American/ Alaskan Native/ Indigenous	6.3
Native Hawaiian/ Pacific Islander	0
White/Caucasian American – Not Hispanic	68.7
Hispanic	0
Other	0
Sexual orientation	
Heterosexual	53.3
Homosexual (including gay or lesbian)	6.7
Bisexual	26.7
Queer	6.7
Other	6.7

Pre/Post-Survey Analysis

**Figure 1:**
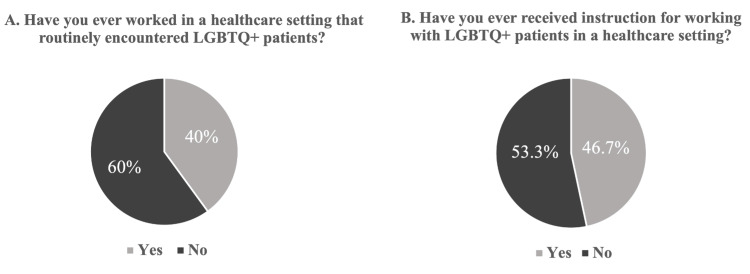
Student exposure in the healthcare setting to (A) LGBTQ+ patients and (B) LGBTQ+ focused education LGBTQ+: Lesbian, Gay, Bisexual, Transgender, Queer

Students were given the opportunity to complete a pre and post survey before and after the workshop. In the pre-survey, 53% of respondents reported never receiving any formal instruction for working with LGBTQ+ patients in a healthcare setting. The majority of participants (60%) indicated they had never worked in a healthcare setting that routinely encountered LGBTQ+ patients. Figure 1 graphically represents this data. 

The students were given a series of statements and asked to rate their confidence and comfortability on a 7-point Likert scale based on a series of 16 statements. A score of 7 indicates ‘extremely comfortable’, 6 ‘comfortable’, 5 ‘somewhat comfortable’, 4 ‘neutral’, 3 ‘somewhat uncomfortable’, 2 ‘uncomfortable’, and 1 ‘extremely uncomfortable’. In the pre-survey, 47% of respondents reported feeling uncomfortable when eliciting a sexual history from a LGBTQ+ patient. The respondents demonstrated a statistically significant increase in comfort and confidence in 12 out of the 16 statements addressed during this workshop. These statements, along with the pre- and post-survey means are displayed in Table 2.

**Table 2:** Assessing student comfort and confidence of LGBTQ+ knowledge pre- and post-workshop Respondents ranked their comfort and confidence between 1 and 7 (7 being ‘extremely comfortable’ and 1 being ‘extremely uncomfortable’) for each statement before and after the workshop. There was a statistically significant increase in comfort in 12 out of the 16 statements. LGBTQ+: Lesbian, Gay, Bisexual, Transgender, Queer

Statement N=15	Pre-Survey Mean	Standard Deviation	Post-Survey Mean	Standard Deviation	p-value	Cohen’s D Value
I feel confident that I can make the changes necessary to address homophobia, biphobia, and transphobia in patient care in my home institution.	4.9	1.6	5.4	1.0	0.2	0.4
I feel confident that I can continue to educate myself on LGBTQ+ healthcare issues and disparities.	6	1.6	6.1	0.5	0.4	0.1
I feel confident that I can teach my colleagues how to decrease LGBTQ+ bias in patient care.	5.5	1.0	5.5	0.8	0.5	0
I am able to identify and acknowledge my privileges.	5.9	1.0	6	0.6	0.3	0.1
I feel competent in utilizing proper LGBTQ+ terminology in healthcare practice.	5	1.2	5.7	0.8	<0.05	0.7
I am comfortable eliciting a sexual history from a LGBTQ+ patient.	4.1	1.3	5.4	0.8	<0.01	1.2
I feel knowledgeable enough to discuss LGBTQ+ specific healthcare with patients.	4.1	1.2	5.6	0.9	<0.001	1.4
I feel competent in the basics of gender affirming care.	4.3	1.4	5.4	0.9	<0.01	1.0
I feel confident in discussing STI concerns specific to LGBTQ+ patients.	4.4	1.2	5.5	1.0	<0.01	1.0
I am able to identify certain health disparities prevalent among LGBTQ+ populations.	4.5	1.4	5.6	0.8	<0.01	1.0
I feel comfortable utilizing proper pronouns with all patients.	5.7	1.1	6.2	0.6	0.1	0.6
I am able to advocate for patients from minoritized backgrounds.	5.3	1.3	6.1	0.3	<0.05	1.0
I am knowledgeable of the factors that affect social and economic circumstances of LGBTQ+ communities.	5	1.2	5.7	0.6	<0.05	0.8
I can explain the concept of minority stress and how these factors contribute to LGBTQ+ health disparities.	4.5	1.4	5.6	0.8	<0.01	1.0
I can discuss ways that language can be used in the medical history to avoid assumptions about a patient's sexual orientation, gender identity, or relationship to other individuals.	4.5	1.4	5.6	1.0	<0.05	0.9
I can describe the terminology, process, and consent challenges for cross-gender hormone therapy.	3.7	1.3	5.2	0.9	<0.001	1.4

After completion of the workshop, none of the participants responded feeling uncomfortable when assessing confidence in LGBTQ+ terminology, eliciting a sexual history, or discussing LGBTQ+ healthcare. All respondents reported that this course would be beneficial to all students pursuing healthcare related degrees.

## Discussion

A two-hour interactive workshop evaluated preclinical students' comfort and confidence when encountering LGBTQ+ clinical health scenarios. Our results demonstrated a significant increase in student comfort and confidence after completion of the workshop. Twelve out of the 16 statements regarding various aspects of LGBTQ+ healthcare demonstrated improvement in the preclinical students’ ability and comfort to advocate for minoritized patients, understand certain disparities within the community, and perform routine healthcare while providing a safe and positive environment.

Additionally, our data demonstrated that most respondents did not have experience or formal training in LGBTQ+ healthcare. Members of the LGBTQ+ community often face unique health inequities and prejudices within our healthcare system which can be further perpetuated by inexperienced providers [13-14]. Increased initiatives to practice LGBTQ+ medicine can improve the healthcare experience for LGBTQ+ individuals and lessen disparities. Patient simulations serve to cultivate effective provider/patient communication and refine clinical skills taught in the classroom to best prepare students for their clinical years and beyond [15]. This has been demonstrated in other studies as well. At the University of New Mexico School of Medicine, third-year clinical students participated in a three-hour LGBTQ+ educational course, with a 30-minute lecture, followed by three, separate standardized patient encounters. Pre- and post-surveys assessing patient comfort levels on taking a sexual history and overall knowledge of sexual practices of LGBTQ+ individuals were collected and demonstrated significant improvement [16]. Similarly, Levy et al. evaluated first-year medical students’ attitudes and knowledge towards LGBT healthcare through a series of case-based discussions. This group’s pre- and post-survey showed significant improvement in attitudes and knowledge of social determinants of health affecting LGBTQ+ individuals [17]. These types of studies highlight both the impact these educational programs have on improving comprehension of LGBTQ+ health topics, and the significance that patient interactions/simulations can have on boosting medical student comfortability. We believe that starting this type of education in the earlier years of medical training, such as preclinical medical students, can provide ample opportunity to create confident and competent future physicians.

Limitations to our study include a small sample size, as well as optional participation in the workshop from pre-clinical students. Initially, this workshop was launched as a pilot program to gauge interest in participation and assess the effectiveness of the course prior to implementing it into a medical school curriculum. Notably, our participant demographics consisted of approximately 40% identifying as being a part of the LGBTQ+ community, much higher than the national average. This may be due to self-selection into the course due to it being optional for first- and second-year medical students; however, it is promising that there was an increase in comfort in approaching LGBTQ+ healthcare topics even within those that identify within the LGBTQ+ community. We hope this workshop is implemented within our core medical school curriculum as a mandatory course to reach a wider audience.

The importance of providing educational opportunities through patient simulations, case-based discussions, lectures, and debriefing allows pre-clinical students to practice and refine clinical skills prior to entering their clinical years. One meta-analysis evaluating the effectiveness of interactive programs on reducing LGBTQ+ healthcare disparities and bias showed increased comfort levels and tolerance after participating in these educational sessions [18]. In our post-survey, all students agreed that the workshop would be both educational and beneficial for all healthcare trainees and providers. Furthermore, none of the participants reported feeling uncomfortable when assessing confidence in LGBTQ+ terminology, eliciting a sexual history, or discussing LGBTQ+ healthcare. Overall, more educational opportunities better prepare healthcare trainees for LGBTQ+ encounters and promote better outcomes. The use of these effective programs can help cultivate a positive experience for the patient, create a safe environment, and combat health disparities.

## Conclusions

Educational opportunities to practice with LGBTQ+ patients during preclinical years can increase medical students' confidence and comfort. Our study suggested that a focused workshop on LGBTQ+ healthcare topics significantly improves preclinical students’ ability and comfort in understanding disparities, variations in need and care, and interactions with LGBTQ+ patients. This workshop can easily be replicated and integrated in a current medical school curriculum to meet the needs of our evolving society while creating a more inclusive space for minority patients.
